# Microstructural properties and enhanced photocatalytic performance of Zn doped CeO_2_ nanocrystals

**DOI:** 10.1038/s41598-017-11074-7

**Published:** 2017-10-02

**Authors:** M. A. Majeed Khan, Wasi Khan, Maqusood Ahamed, Abdulaziz N. Alhazaa

**Affiliations:** 1 King Abdullah Institute for Nanotechnology, King Saud University, Riyadh, 11451 Saudi Arabia; 2 Department of Physics, Aligarh Muslim University, Aligarh, 202002 India; 3 Physics and Astronomy Department, College of Science, King Saud University, P. O. Box 2455, Riyadh, 11451 Saudi Arabia

## Abstract

The microstructural, optical and photocatalytic properties of undoped and 5% Zn doped CeO_2_ nanocrystals (NCs) have been explored through various analytical techniques, viz. powder x-ray diffraction (PXRD), x-ray photoelectron spectroscopy (XPS), transmission electron microscopy (TEM), UV-visible, Raman and photoluminescence (PL) spectroscopy. XRD data analysis revealed face centred cubic (FCC) crystal symmetry of the samples with average crystallite size in the range of 19–24 nm. XPS results confirmed that the Zn ions exist in +2 states and successfully incorporated into the CeO_2_ matrix. Internal structure and morphology observed by TEM exhibited almost uniform cubical shape of the particles of average size ~20–26 nm. The enegy bandgap of undoped and Zn doped CeO_2_ NCs had a direct transition of 3.46 eV and 3.57 eV respectively as estimated by the optical absorption data. The increase in the bandgap revealed blue shift of absorption edge due to the quantum confinement effects. The NCs exhibited an inherent luminescence emission peak at ~408 nm in PL spectra. Improvement in the photocatalytic activity was observed for Zn incorporated sample attributed to the enhanced light absorption or/and fall in charge recombination rate between CeO_2_ and Zn.

## Introduction

During the last few decades, nanoparticle research has been the most studied branch of science and technology due to its numerous applications in various sectors. Nanostructured materials/nanomaterials have diversity of potential uses in optical, electronic and biomedical applications. The possible reason of their different applications can be structural features lies from atomic size to bulk materials. On the nanometric scale, physical and chemical properties of the materials are quite different from those of bulk materials. Another fundamental cause is that the nanomaterials have a much greater surface to volume ratio, spatial confinement and high surface energy. For these reasons, nanoparticle shapes are of great interest because different shapes have a distinct surface-to-volume, as well as other characteristics. More recently, room temperature ferromagnetism has also been noticed at nanoscale in wide bandgap diluted magnetic semiconductors, for exampleTiO_2_, ZnO, SnO_2_, In_2_O_3_, CeO_2_ etc. The observed ferromagnetism in these nanomaterials has been attributed to oxygen vacancies and low dimensionality^1^. In contrast, Coey *et al*. suggested the magnetism in CeO_2_ nanoparticles by proposing a new model based on giant orbital paramagnetism (GOP) accompanying with the collective response of electrons in coherent domains to applied magnetic field^2^. Nevertheless, the source of ferromagnetism in these systems is not yet well understood^3^.

In this class of nanomaterials, cerium oxide or ceria (CeO_2_) has been acknowledged for its various scientific, technological and biomedical potential applications^4^. CeO_2_ is the most reactive rare earth oxide having face centred cubic (FCC) crystal structure of fluorite type. It has attracted lots of interest in the scientific community owing to its large energy gap (E_g_ ~ 3.19 eV) and extraordinary dielectric properties (ε = 24.5)^5^. Due to these outstanding properties, CeO_2_ is very valuable for evolved technological, energy and environmental applications^6^ like inorganic UV filters^7^, solar cells^8^, electrochromic smart windows^9^, photocatalytic systems^10^, solid oxide fuel cells^11,12^, glass-polishing materials^13^, low temperature water-gas shifts (WGS) reaction^14^, gas sensors^15^, optics^16^, electrochromic thin film applications^17^, environmental chemistry, medicine^18^, and ultraviolet light blockers^19^, etc.

Nowadays, attention has been paid to the doped cerium oxide in low dimensions with metal ions that display many novel and exciting properties. Recently, Xia *et al*.^20^ prepared Mn incorporated CeO_2_ nanorods via a facile composite hydroxide mediated (CHSM) approach and they concluded that the synthesised nanorods are suitable for potential applications in the building of photovoltaic devices and photocatalytic activity. However, to improve the physical properties of CeO_2_, we have chosen Zn ions because it is one of the common and highly stable dopants. Ramasamy *et al*.^21^ recently synthesized and investigated the physical properties of Zn doped CeO_2_ with different concentration of Zn ion. They suggested enhancement in the photoluminescence and photocatalytic activity on Zn doping. Despite of various useful characteristics, one of the major drawbacks of CeO_2_ lies in its wide energy gap that makes it active only in ultraviolet radiation and is not in visible light. To resolve this problem, many notable efforts have been made in this direction by altering the CeO_2_ system and improving its photocatalytic performance in visible radiation. There are different ways of conducting the synthesis of small-scale particles, especially oxides and mixed state of oxides suitable for catalytic applications. Recently, various approaches were explored to synthesis nanocrystalline powder of CeO_2_, including spray pyrolysis, electrospinning synthesis, gas condensation, as well as precipitation from oxalate, carbonate, peroxide, hydroxide, polymeric precursors, and organometallic decomposition^22–33^. But we have obtained these nanocrystals (NCs) from commercially available source to exclude the possibility of impurities and irreproducibility in the results.

We believe this the first report on the structural, thermal, optical and photocatalytic properties of monodisperse Zn incorporated CeO_2_ NCs. Subsequently, it is exciting to explore the above mentioned properties of nanoscale Zn-CeO_2_ crystals. Apart from the potential applications of these systems in diverse fields, it will also provide useful information from the basic physics point of view.

## Results and Discussion

### Structural Analysis

**Figure 1:**
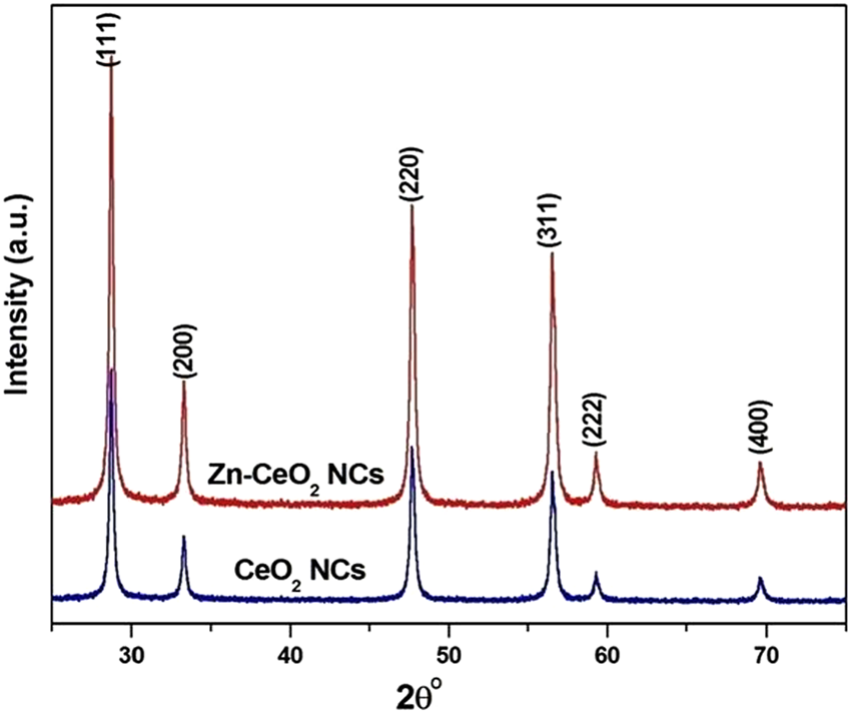
XRD patterns of undoped and Zn-doped CeO_2_ nanocrystals.

**Figure 2:**
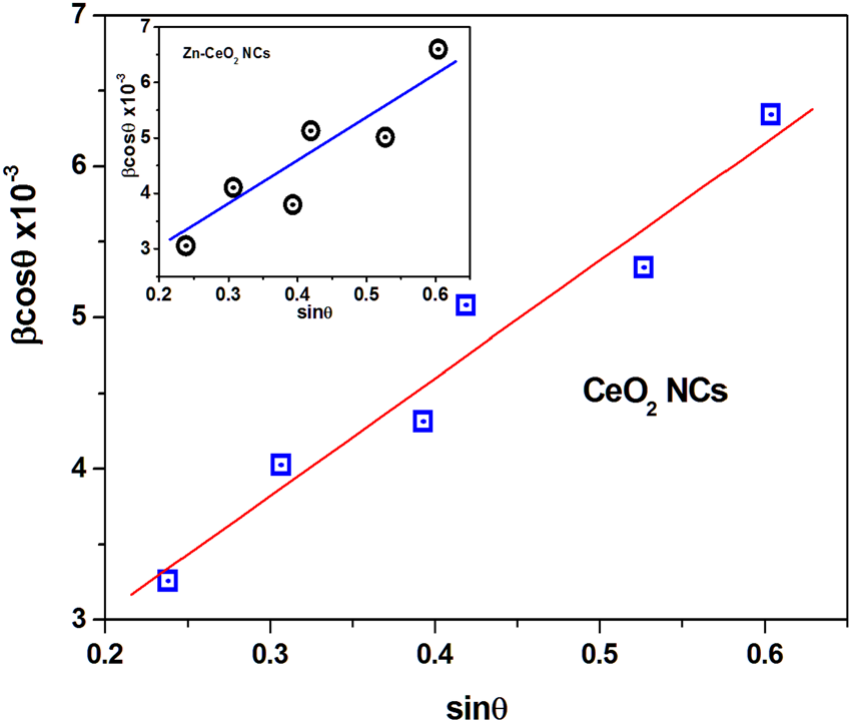
Williamson-Hall plot for CeO_2_ nanocrystals and inset shows same for Zn-doped CeO_2_ nanocrystals.

To identify the phase, purity and crystallinity of undoped and Zn doped CeO_2_ NCs, powder XRD technique was used and the obtained diffraction patterns are presented in Fig. 1. It is clear that the Bragg’s peaks appeared at 2θ values 28.6^o^, 32.8^o^, 47.5^o^, 56.3^o^, 59.24^o^ and 69.56^o^ correspond to the (111), (200), (220), (311), (222), and (400) crystal planes respectively﻿. All peaks in the diffraction patterns are indexed to the pure cubic fluorite structure of cerium oxide^34^. Moreover, no additional peaks related to other elements were detected, signifying the single phase, highly pure nature and proper substitution of Zn ion at Ce site in the NCs. The intensities and position of the peaks are in close agreement with the values given in the literature^35^. The lattice parameters (*a* = *b* = *c*) estimated from the XRD patterns are found to be 5.37121 and 5.37550 Å for undoped and Zn doped CeO_2_ samples, respectively, that are consistent with earlier reported results^21^ and slightly less than the bulk form of ceria (*a* = 5.41134 Å, JCPDS 34–0394). The larger broadening of the diffraction peaks clearly indicate the nanoscale size of the crystallites. In addition, Scherrer’s equation^36^ is used to confirm low dimensions of the crystallites in the samples and are found to be 24 nm for undoped and 19 nm for Zn doped CeO_2_ NCs estimated using (111) crystallographic plane. The relationship between the crystallite size and lattice strain on the broadening of the XRD peaks are studied by Williamson–Hall (W-H) analysis^37^:

1 $$\beta cos\theta =\frac{C\lambda }{D}+4\varepsilon sin\theta $$

where β, D, ε are the full width at half-maximum of the peak in radian, average crystallite and induced strain respectively. C represents shape factor correction and equal to 1. According to the above equation, crystallite size and strain are estimated by plotting βcosθ as a function of sinθ as shown in Fig. 2. The linear fit of the data is used to calculate the microstrain from the slope of the straight line and crystallite size by the interception on y-axis. It is known that the reduction in particle/crystallite size can significantly enhance lattice strain in the sample. However, position and broadening of the XRD peak can be influenced by the variety of factors, including crystallite size, shape, inhomogeneity and strain. For the present nanocrystals of undoped and Zn doped CeO_2_, the lattice strain and average value of the crystallite size are estimated as 1.37 × 10^−3^, 5.97 × 10^−3^ and 26 nm, 20 nm respectively. Therefore, it is clear that the decrease in the crystallite size increases the induced lattice strain in the doped sample, which makes it thermally stable material. In addition, the estimated crystallite size revealed nanostructures of both the samples.

### XPS Studies

**Figure 3:**
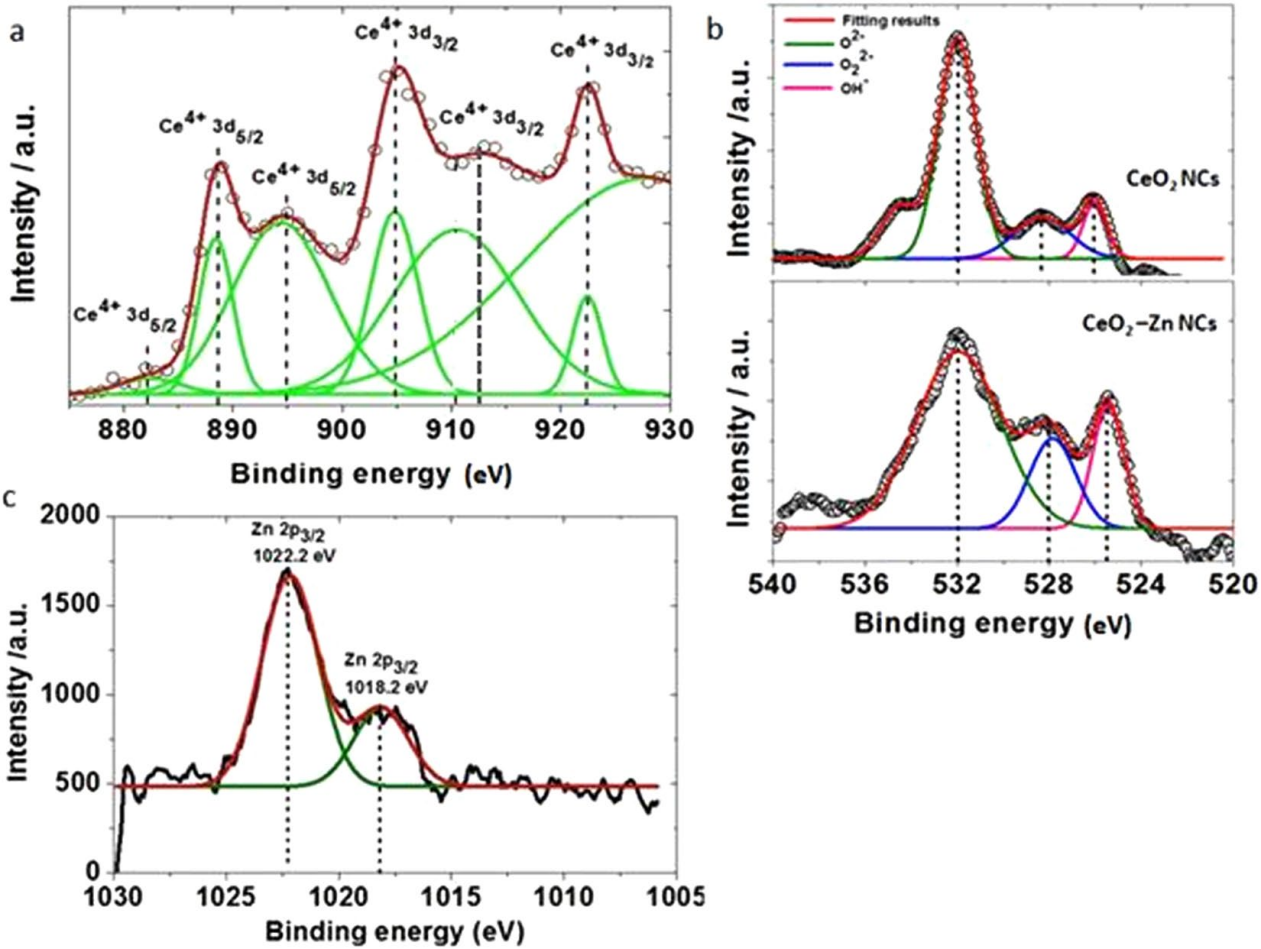
Experimental and peak fitting XPS spectra of CeO_2_ (**a**) Ce 3d level (**b**) O 1 s level for pure and Zn doped CeO_2_ NCs (**d**) Zn 2p_3/2_.

XPS was employed to investigate the quantitative analysis such as elemental composition and electronic state of Zn doped CeO_2_ NCs. Wide scan XPS spectrum exhibits a very clear CeO_2_ features and presence of an additional Zn1s signal in the range of 250–440 eV, which indicates that the Zn particles had been successfully incorporated into the CeO_2_ matrix (Fig. not shown here). The peaks appeared at 881.9, 888.7, and 894.9 eV correspond to the components of Ce3d_5/2_^38^, while the remaining signals of Ce 3d_3/2_ components could be seen at 905.1, 912.8 and 921.9 eV (Fig. 3a). The peak position of oxygen is slightly shifted due to the incorporation of Zn ion in CeO_2_ compared to undoped CeO_2_. Figure 3(b) displays representative typical XPS spectra in the O1s region for pure and Zn doped CeO_2_ samples. The main XPS peak for O1s is shown around 531.9 eV and may have originated from the oxygen atoms in the lattice^39^, whereas the other peaks observed in the range of 528.1 eV and 525.5 eV because of chemisorbed oxygen caused by surface hydroxyl, that corresponds to the O-H bonds^40^. Moreover, the XPS spectrum of Zn 2p level in Fig. 3(c) revealed two prominent peaks at the binding energies of 1022.2 and 1018.2 eV for the presence of oxidative and reductive zinc respectively, refer to Zn 2p_3/2_.﻿These results support that the Ce and Zn ions exist﻿﻿ in 4+ and 2+ oxidation states respectively.

### Microscopic Studies

**Figure 4:**
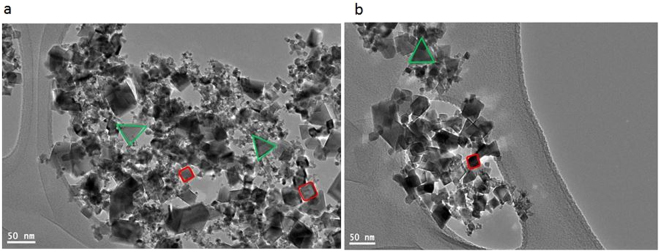
TEM images of (**a**) CeO_2_, and (**b**) Zn-doped CeO_2_ nanocrystals.

**Figure 5:**
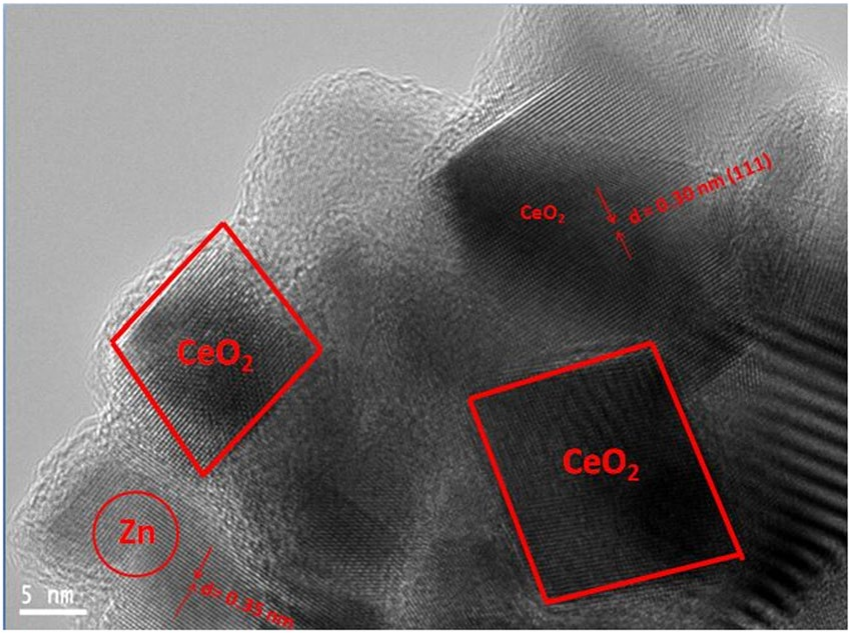
HRTEM image of Zn-doped CeO_2_ nanocrystals.

**Figure 6:**
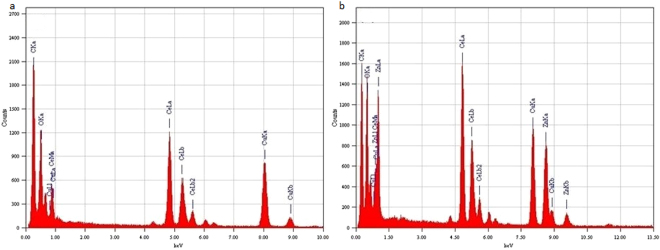
EDX patterns of (**a**) CeO_2_, (**b**) Zn-doped CeO_2_ nanocrystals.

To elucidate the crystal structure, growth direction, and elemental composition of the NCs, transmission electron microscopy (TEM) and high-resolution TEM (HRTEM) measurements have been performed. TEM images of undoped and Zn-doped CeO_2_ NCs are shown in Fig. 4(a,b) respectively. These images exhibit combinations of the cubes and partially spherical shapes of average size in the range of 20–26 nm and these results are well matched with the particle size estimated from the XRD data using Debye-Scherrer equation. In addition, crystalline nature of the doped NCs further confirmed by the HRTEM as presented in Fig. 5 and then interplanar spacing (d) is measured as ~2.52 Å. This value of the spacing corresponds to (111) lattice plane of an *fcc* fluorite type CeO_2_ structure (JCPDS: 34-0394). Moreover, these results are also agreed well with the XRD data analysis. EDX spectra of the NCs as shown in Fig. 6(a,b), yielded peaks corresponding to the investigated elemental composition (Ce, Zn, O), but also a signal from the carbon-coated copper grid appears on which the samples were placed (C, Cu). The Cu-peaks around 8 keV arising from the interior of the TEM and cannot be used to identify Cu in the sample.

### Thermogravimetric analysis

**Figure 7:**
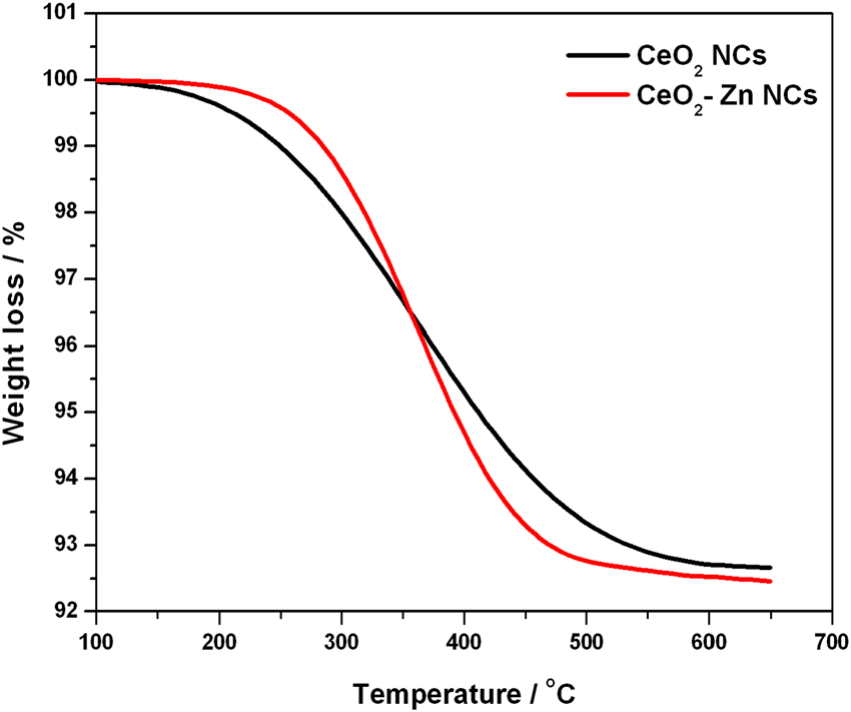
TGA spectra of the undoped and Zn doped CeO_2_ NCs.

Thermogravimetric analysis (TGA) was used to study the thermal stability of undoped and Zn doped CeO_2_ NCs by observing the change in mass with the variation of temperature and time under controlled atmospheric conditions. TGA measurement is generally performed in a helium or argon (inert gas) atmosphere, and the loss/gain in weight is monitored by increasing the temperature. In this work, measurement is performed at a heating rate of 10 °C/min in the range from ambient temperature to 650 °C and TGA plots are shown in Fig. 7 for both samples. These curves exhibit that the weight start to decrease at 125 °C and ended at 600 °C (Fig. 7) for both samples. The weight loss at first stage originates from the evaporation of water that absorbed by the samples at ambient temperature, while the second one is due to formation of cubic fluorite CeO_2_ phase^41^.

### Raman Analysis

**Figure 8:**
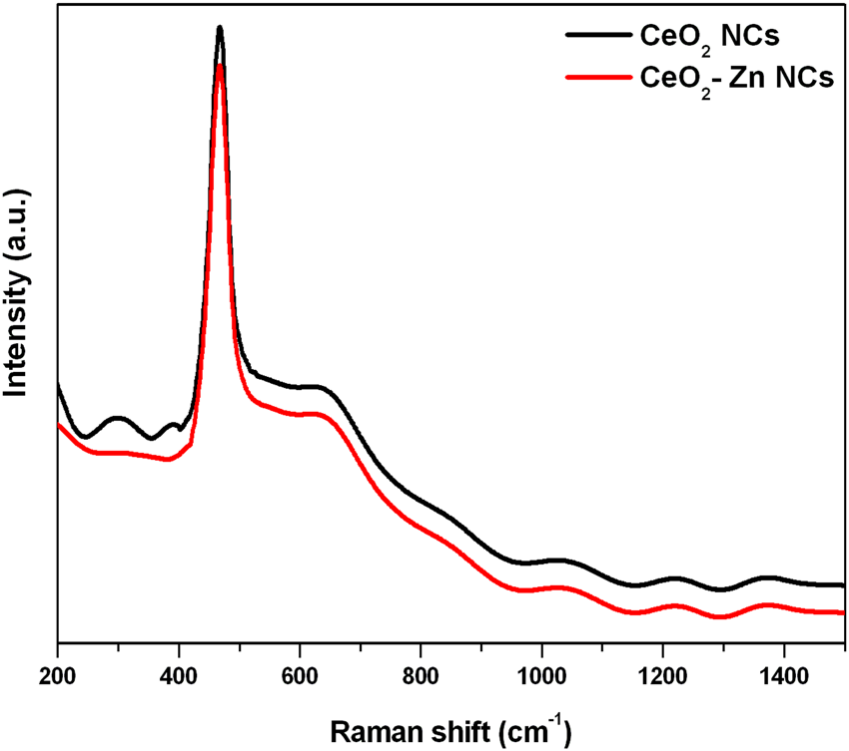
Raman spectra of undoped and Zn-doped CeO_2_ nanocrystals.

To study the vibrational, rotational and other low-frequency modes in the samples, Raman spectroscopy technique has been employed. Using this technique, significant modifications in the band frequencies can be observed easily if any change in lattice parameters and chemical environment occurs^42^. The Raman spectra of undoped and Zn doped CeO_2_ NCs have been measured in the spectral range of 200–1500 cm^−1^ as shown in Fig. 8. We have observed a Raman active band F_2g_ at ~468 cm^−1^ that may be due to the fluorite type structure of CeO_2_^43^. This again validates *fcc* crystalline structure of the NCs and hence the vibrational mode is nearly independent of the ionic mass of CeO_2_ by the movement of O atoms^41,44,45^. The other bands at 643, 1044, 1221 and 1377 cm^−1^ are due to the presence of the oxygen vacancies created by the charge compensation for the defects induced by the incorporation of other metal cations into the ceria lattice^46^. The formation of CeO_2_ is well agreed with the XRD results.

### FTIR Spectroscopy

**Figure 9:**
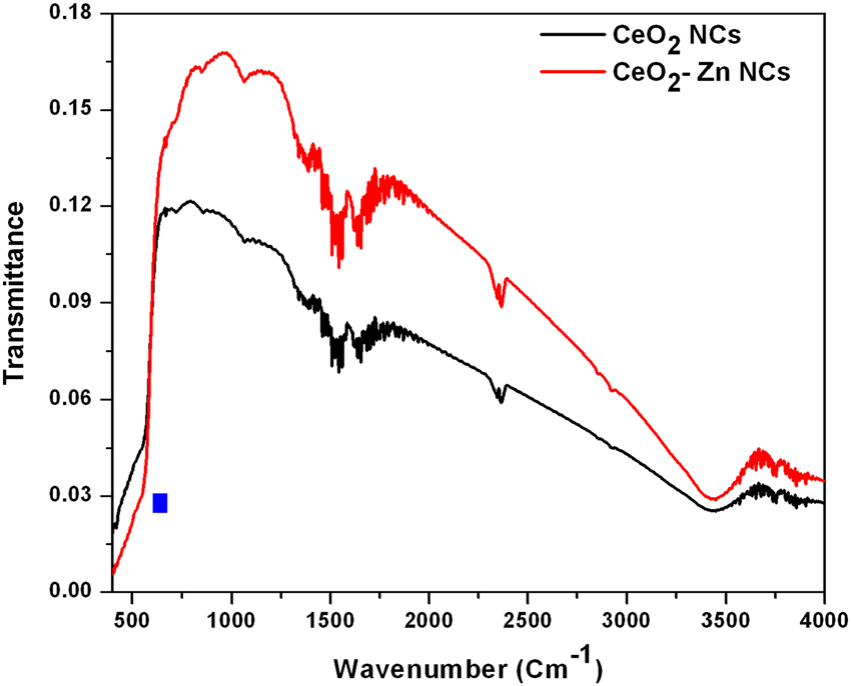
FTIR spectra of undoped and Zn-doped CeO_2_ nanocrystals.

Infrared spectroscopy is used to identify the functional groups and to study the vibrational signatures of the atoms/molecules. The FTIR spectra of CeO_2_ and Zn doped CeO_2_ NCs are shown in Fig. 9 in the wavenumber range of 4000 to 400 cm^−1^. This figure indicates the well defined characteristic absorption bands appeared at 548, 1065, 1625, 2368 and 3437 cm^−1^, confirmed the existence of pure CeO_2_ phase^47^. Moreover, the bands below 700 cm^−1^ (i.e. in the range of 400–650 cm^−1^) correspond to the stretching frequency of Ce-O^48^. However, absorption peaks located at 1065 and 1625 cm^−1^ are attributed to moisture or absorbed water and CO_2_ molecules, that are generally absorbed by the nanomaterial’s surrounding environment due to their large surface-to-volume ratio^49^. Furthermore, a broad absorption band occurred at 3437 cm^−1^ is ascribed to the O-H stretching mode and bending associated with water molecules on the CeO_2_ surface.

### Optical Studies

**Figure 10:**
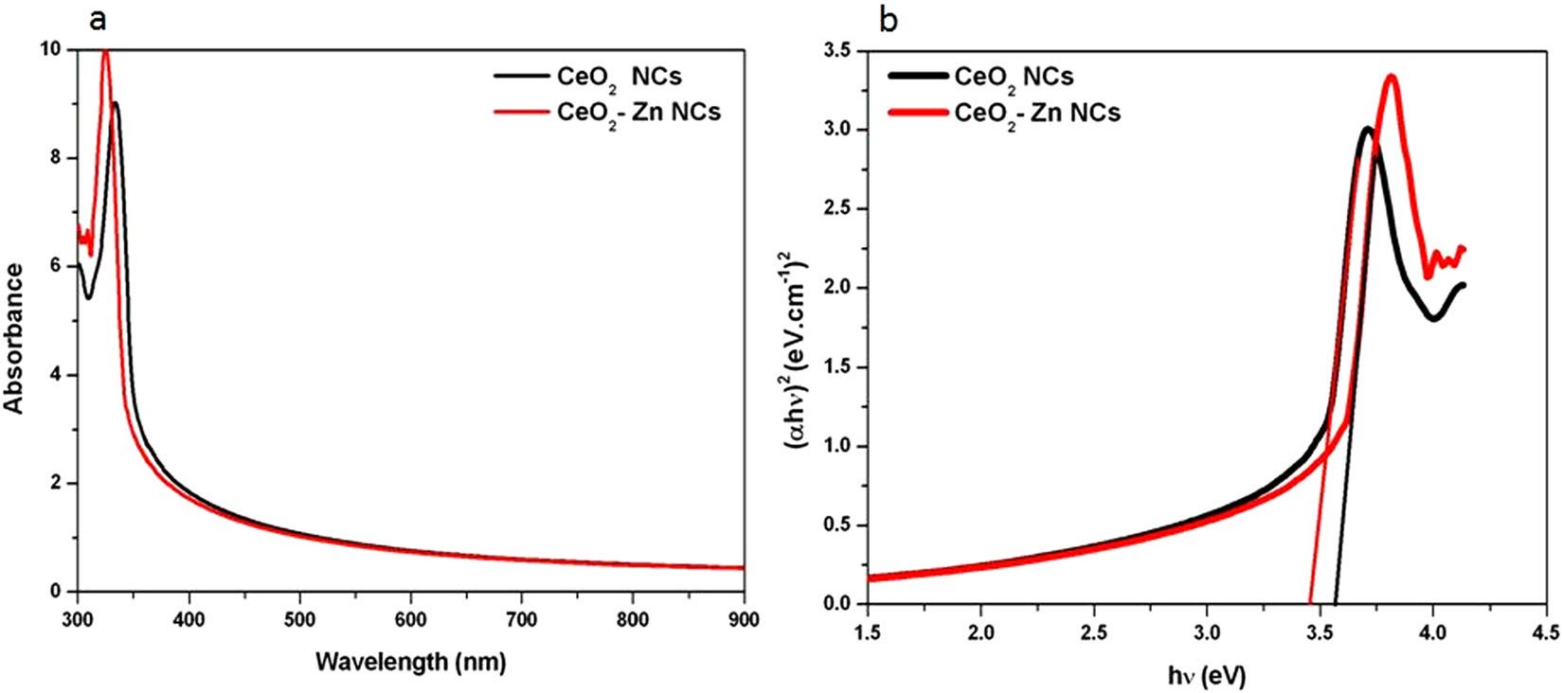
(**a**) Optical absorption spectra and (**b**) (αhν)^2^ versus hν for undoped and Zn-doped CeO_2_ nanocrystals.

In order to investigate the effect of Zn ion doping on the optical band-gap of the CeO_2_ NCs, UV-visible absorption study was carried out. The absorption spectra for undoped and Zn doped CeO_2_ NCs were determined in the spectral range of 300–900 nm as presented in Fig. 10(a). A strong absorption peak is observed at 335 nm for undoped CeO_2_ nanocrystals, that shifted towards the lower wavelength (324 nm) by the incorporation of Zn ions at Ce site. Here, it is clear that the absorption edge of both NCs is blue-shifted compared to bulk CeO_2_. The peak shifted towards the lower wavelength side in the doped sample indicated a change in the electronic band structure. Using absorption data, the energy gap (E_g_) of the samples was estimated from the following equation^50^:

2 $${(\alpha h\nu )}^{2}=A({\rm{h}}\nu -{{\rm{E}}}_{{\rm{g}}})$$

where α is the absorption coefficient, hν is the photon energy, and A is a constant that does not depend on photon energy. The energy band gap can be estimated from the straight line on the x-intercept, as shown in Fig. 10(b). It is observed that the band gap of CeO_2_ nanocrystals is 3.46 eV and this value increased to 3.57 eV for Zn doped sample. These values are higher than that of the bulk CeO_2_ (E_g_ ~ 3.19 eV)^6,51^. Our results are in close agreement with the previous reports^52,53^. The band-edge absorption of nanoscale semiconductor materials is mainly dependent on two factors: the quantum size and interface effects. Generally, the quantum size effect leads to a blue shift, that predicts an increase of the band-gap value with decreasing particle size, while the interface effects induce a red shift. In this work, the quantum size effect should be responsible for the variation of the absorption band edge. The size dependence energy band gap can be estimated using the following expression

3 $${E}_{g(nano)}={E}_{g(bulk)}+\frac{{\rm{\pi }}{h}^{2}}{2{R}^{2}}(\frac{1}{{m}_{e}}+\frac{1}{{m}_{h}})-\frac{1.8{e}^{2}}{{\rm{\varepsilon }}R}$$

where E_g_ (bulk) is the band gap of bulk material (3.19 eV), R is the nanoparticles radius, m_e_ and m_h_ are the electron and hole effective masses respectively, and it is considered as (m_e_ = m_h_ = 0.4 for CeO_2_), ε(24.5) as the permittivity for CeO_2_^6,51^; *h* is Planck’s constant and e is the electronic charge. It is clear from the above equation that the energy band gap (E_g_) increases when the particle size is decreases. As mentioned above, the blue shift occurs due to quantum confinement and the concentration of Ce ions in the grain boundary decreases by the doping, so the band-gap energy increases.

### Photoluminescence Studies

**Figure 11:**
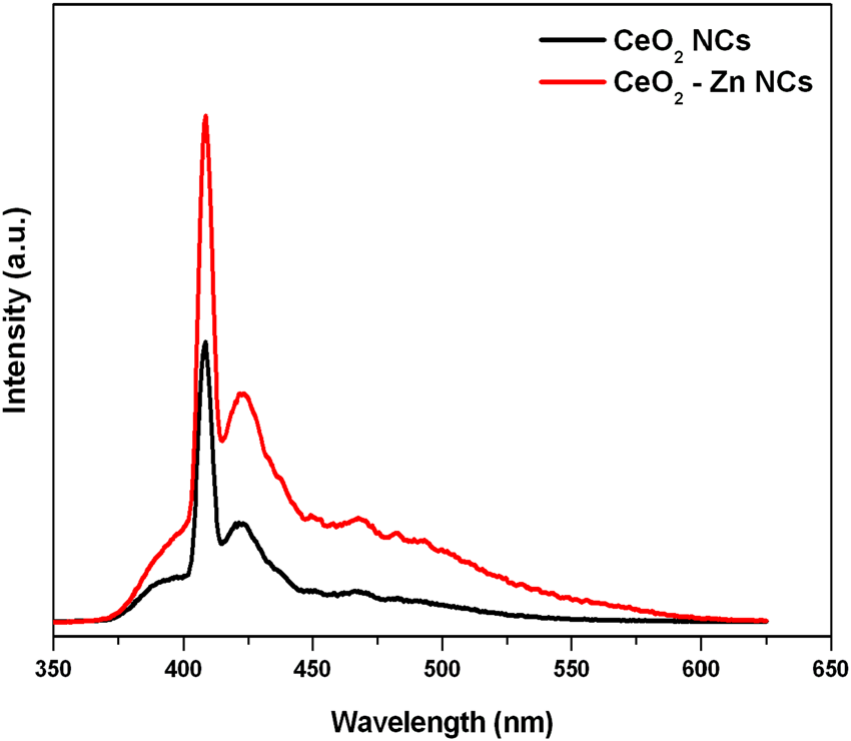
Photoluminescence (PL) spectra of undoped and Zn-doped CeO_2_ nanocrystals at room-temperature.

Photoluminescence (PL) spectroscopy is a powerful tool to investigate the electronic structure of materials. Like other optical techniques, it is also fast and non-destructive. PL spectra of undoped and Zn doped NCs are recorded with an excitation wavelength of 325 nm and displayed in Fig. 11. The observed emission peak in the spectra centred at 408 nm can be attributed to the intrinsic luminescence of CeO_2_. The bands appeared in the range of 400–500 nm, suggested the relative oxygen vacancy in CeO_2_ NCs^54^. This phenomenon can be explained on the basis of transfer of charge from the 4f band to the valence band of the CeO_2_ in both samples^55^. It is attributed to the electron transition from the Ce 4f conduction band with holes in to O 2p valence band, while the defect energy levels are located in the site between the Ce4f and O2p bands, resulted in wider emission bands. Therefore, it confirmed that the emission in studied NCs originated by the transition from the cerium 4f band to the oxygen 2p band (valence band) in CeO_2_.

### Photocatalytic activity

**Figure 12:**
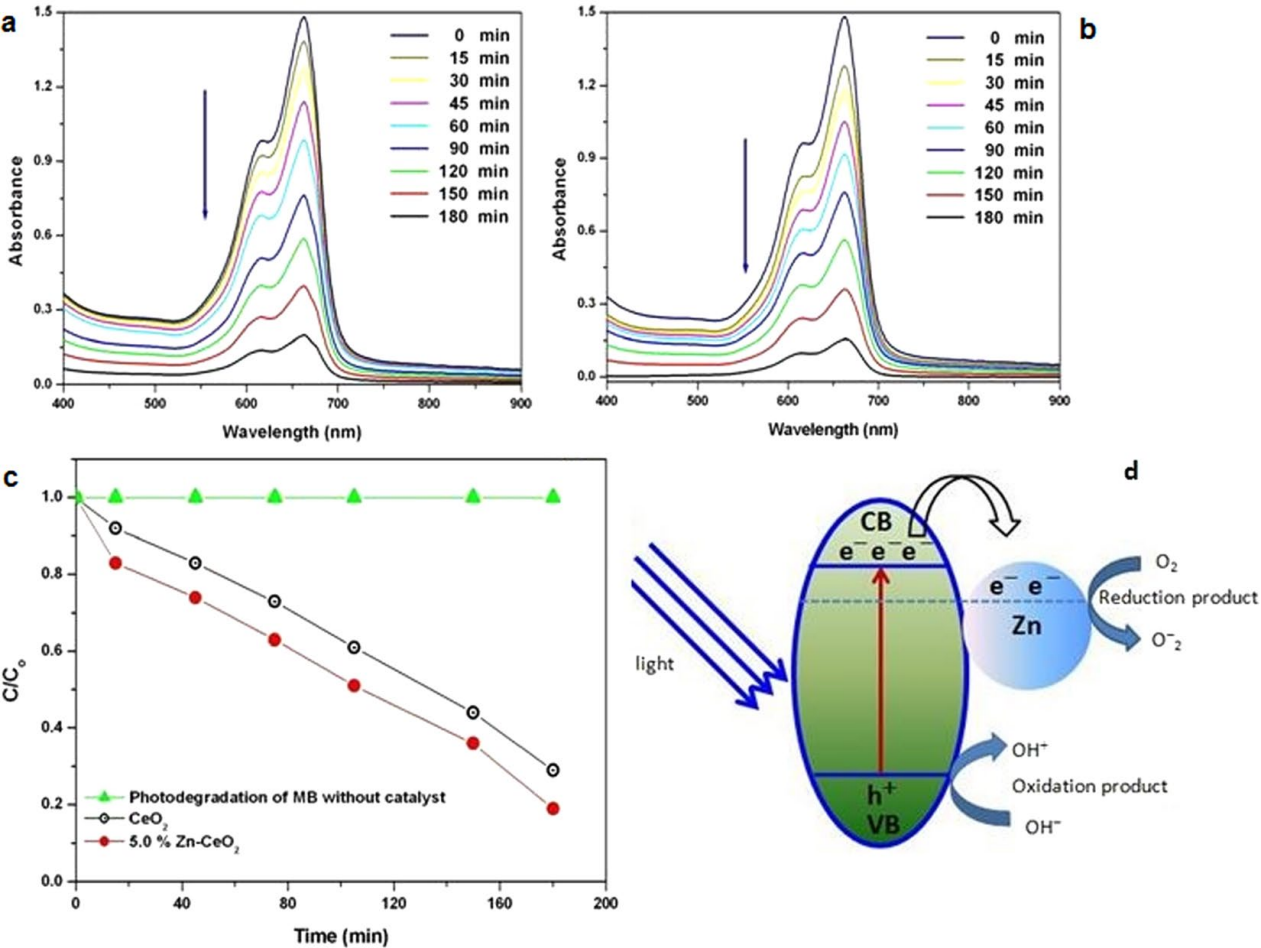
Time-dependent UV-vis absorption spectra of the methylene blue (MB) aqueous solution in the presence of (**a**) CeO_2_, (**b**) Zn doped CeO_2_ as a function of irradiation time. (**c**) Plots of C/C_o_ versus irradiation time. (**d**) A possible photocatalytic mechanism of MB in Zn doped CeO_2_ catalyst.

The photocatalytic degradation of methylene blue (MB) dye under visible light irradiation by using undoped and Zn substituted CeO_2_ NCs were evaluated and the observed absorption spectra are presented in Fig. 12(a,b). It is clearly shown in the figure that the characteristic absorption peak of MB at about 664 nm decreases gradually with increasing exposure time from 0 to 180 min and almost disappears after 180 min of irradiation time which indicates that the MB dye has almost degraded. In addition, a blank experiment is also performed to ensure that the catalyst or the visible radiation is necessary for photo-conversion reaction (Fig. 12(c)). The photocatalytic degradation of MB dye by CeO_2_ and Zn doped CeO_2_ nanocrystals photocatalysts under visible irradiation fits by the pseudo-first-order equation and their kinetics may be expressed as follows^56^

$$ln\frac{C}{{C}_{o}}=kt$$

where C_o_ and C stand for the initial concentration and concentration at various contact times (t) respectively. ‘k’ and ‘t’ are the reaction rate and reaction time. The photocatalytic performance of the NCs are examined by plotted (C/C_o_) as a function of time for MB dye and shown in Fig. 12(c). This figure infers that the degradation rate of MB in the presence of Zn doped CeO_2_ nanocrystals is 78.1% after 180 min of irradiation time that is slightly higher than that of pure CeO_2_ nanocrystals (70.2%). The probable reasons for the higher activity of the doped CeO_2_ photocatalyst as compared to undoped could be the attachment of Zn nanoparticles at the surface of CeO_2_ nanocrystals. This causes the electron sinks and hinder recombination of the photoinduced electrons and holes resulting in better charge separation than CeO_2_, and thus a better inhibition of photoinduced electron recombination with photoinduced hole pairs is occurred^57–60^. This might be liable for enhanced photocatalytic activity of doped Zn/CeO_2_ photocatalyst^61,62^.

In order to further confirm the possible degradation mechanism of the dye in addition to Zn/CeO_2_, the relative energy positions of the conduction band (CB) and valence band (VB) were investigated. The position of these bands in CeO_2_ can be estimated by the following relations^63^:

4 $${E}_{CB}(Ce{O}_{2})=\chi (Ce{O}_{2})-{E}^{C}-\frac{{E}_{g}}{2}$$

5 $${E}_{VB}(Ce{O}_{2})={E}_{g}-{E}_{CB}(Ce{O}_{2})$$

where χ is electronegativity of the sample (Here, χ = 5.56 eV for CeO_2_), E^c^ and E_g_ represent free electrons’ energy on the hydrogen scale (~4.5 eV) and the energy gap of CeO_2_ (~3 eV) respectively. The estimated values of the CB and VB positions in CeO_2_ NCs are −0.67 and 2.79 eV respectively. Accordingly, the CB and VB edge positions in Zn doped CeO_2_ NCs are found to be −0.73 and 2.85 eV respectively. The values of VB positions for pure and Zn doped CeO_2_ NCs are higher than the water reduction potential and indicate that these materials are good and may a potential candidate for photocatalytic O_2_ production.

The coupled semiconductor materials have two different band-edge potential and energy-levels which play a significant role in determining the flowchart of photoexcited charge carriers in these semiconducting materials. The photocatalytic reaction mechanisms for oxidation of MB dye by undoped and Zn doped CeO_2_ nanocrystals are presented in Fig. 12(d). In the visible light illumination, electrons start to excite from the valence band to the conduction band by producing the equal number of holes in the valence band, if energy of the incident photons (hν) is greater than or equal to band gap. Photocatalytic activity of the CeO_2_ NCs is improved by Zn doping ascribed to the photo absorptions spreading to the visible region and minimizing the electron-hole recombination rate. Thus, the efficiency of photogenerated electron-hole in Zn/CeO_2_ could be higher than those of pure CeO_2_ nanocrystals.

## Conclusions

In summary, undoped and Zn doped CeO_2_ NCs have been studied to explore their structural, optical and photocatalytic properties through various techniques like XRD, XPS, TEM/HRTEM, Raman, UV-visible and photoluminescence (PL) spectroscopy. Average grain size estimated from the Williamson–Hall analysis, Debye–Scherrer equation and TEM image analysis are very close to  each other. The analysis of XRD data and Raman spectroscopy established highly pure, single phase and cubic fluorite structure of the samples, and slightly increase in lattice parameter was also observed with the reduction in crystallite size. The effect of bandgap tuning was observed in UV-visible absorption spectra of CeO_2_ NCs with Zn incorporation. A blue shift in the absorption was observed in CeO_2_ NCs compared to its bulk counterpart, as well as for Zn doped CeO_2_ sample. It was also demonstrated that the crystallinity and luminescence properties of the nanocomposite increased significantly after Zn doping. Finally, Zn doped CeO_2_ NCs exhibit superior photocatalytic performance compared to undoped NCs for the degradation of methylene blue dye under visible light.

## Experimental Details

### Materials and method

Zn-doped CeO_2_ nanocrystals with atomic contents of 5 wt% have been prepared by standard mechanical milling followed by sintering process. The objectives of the ball milling process include the homogeneous mixing or blending, and change in particle shape which is not possible using hand grinding. In a typical experiment, commercial available Zn and CeO_2_ nanopowders of high purity were used as starting material. A high-energy planetary ball mill (PM 100, Retsch, Germany) was employed for milling purpose at room temperature with a ball to powder ratio 10:1. The speed of the milling was maintained at 400 rpm for 6 h. After obtaining well-proven mixing of the precursors, the mill was stopped and the powder was washed with distilled water and then with ethyl alcohol followed by drying.

### Samples Characterizations

The microstructure and purity of the samples in the form of NCs were investigated via powder x-ray diffraction (PXRD) technique using Cu-K_α_ radiations of wavelength 1.54060 Å. XRD data were collected in the 2θ range of 20 to 80^ο^ at a scan rate 0.02°/s. X-ray photoelectron spectroscopy (XPS) was employed to confirm chemical compositions and oxidation states of the samples using an ESCA model VG 3000 system with monochromatic Mg K_α_ line (1253.6 eV) radiation. The UV-visible absorption spectra of undoped and doped sample NCs was measured in the 300 nm to 900 nm wavelength range at a resolution of 0.5 nm by a Shimadzu UV-1800 spectrometer.

The size of the nanocrystals and Zn doping modifications in the structure were further characterised by transmission electron microscopy (FETEM, JEOL, JEM-2100 F) having energy dispersive x-ray spectroscopy (EDS) analysis and high-resolution TEM (HRTEM, JEOL 2100 F). Studied NCs were also characterized by micro-Raman and micro-PL spectroscopy through Horiba Raman/PL system (IY-Horiba-T64000) having continuous wave laser operating at a 325 nm with an excitation source of 200 cm^−1^ to 1000 cm^−1^.

### Photocatalytic degradation

To verify the photocatalytic activity of undoped CeO_2_ and Zn doped CeO_2_ NCs, decomposition of methylene blue (MB) dye was performed under visible light source of 400 W sodium lamp, Philips, with wavelength range 300–800 nm. For the purpose, 500 mg of each photocatalysts (NCs) were mixed in 500 mL MB solution of initial concentration 3 × 10^−5^ mol/L in a cylindrical vessel (600 ml) and then the solution was magnetically stirred for 30 min in dark to achieve adsorption or desorption equilibrium before light illumination. During the process of the reaction, about 2 mL suspensions were taken and centrifuged and then filtered to remove the residual catalyst particulates for analysis. Afterward, the solution was analyzed using visible spectrophotometer at the characteristic wavelength, from which the degradation yield was calculated. The whole experimental process was conducted under N_2_ bubbling at the flow rate of 80 mL/min.

The degradation efficiency (η) of MB dye was evaluated by the following equation

$${\rm{\eta }}=(1-\frac{{\rm{C}}}{{{\rm{C}}}_{{\rm{o}}}})\times 100$$

where C_o_ gives the initial concentration of the solution and C is the concentration after different light irradiation.
